# A clinicohaematological prognostic model for nasal-type natural killer/T-cell lymphoma: A multicenter study

**DOI:** 10.1038/s41598-019-51522-0

**Published:** 2019-10-18

**Authors:** Khee Ming Tan, Burton Chia, Jing Quan Lim, Lay Poh Khoo, Chee Leong Cheng, Leonard Tan, Eileen Poon, Nagavalli Somasundaram, Mohamad Farid, Tiffany Pooi Ling Tang, Miriam Tao, Daryl Ming Zhe Cheah, Yurike Laurensia, Jane Wan Lu Pang, Tammy Song, Jing Tan, Dachuan Huang, Seok Jin Kim, Won Seog Kim, Choon Kiat Ong, Soon Thye Lim, Jason Yongsheng Chan

**Affiliations:** 1 Lee Kong Chian School of Medicine, Nanyang Technological University, Singapore, Singapore; 2 Lymphoma Genomic Translational Research Laboratory, Division of Cellular and Molecular Research, National Cancer Centre Singapore, Singapore, Singapore; 3 Division of Medical Oncology, National Cancer Centre Singapore, Singapore, Singapore; 4 Department of Anatomical Pathology, Singapore General Hospital, Singapore, Singapore; 5 SingHealth Duke-NUS Blood Cancer Centre, Singapore, Singapore; 6 Samsung Medical Centre, Seoul, South Korea; 7 Genome Institute of Singapore, A*STAR, Singapore, Singapore; 8 Duke-NUS Medical School, Singapore, Singapore; 9 Cancer Science Institute of Singapore, National University of Singapore, Singapore, Singapore

**Keywords:** T-cell lymphoma, T-cell lymphoma

## Abstract

Extranodal NK/T-cell lymphoma, nasal type (NKTL) is an aggressive type of non-Hodgkin lymphoma closely associated with Epstein-Barr virus and characterized by varying degrees of systemic inflammation. We aim to examine the prognostic significance of peripheral blood neutrophil-lymphocyte ratio (NLR) in patients with NKTL. Therefore, we conducted a retrospective review of 178 patients with biopsy-proven NKTL from the National Cancer Centre Singapore and Samsung Medical Center, South Korea. Using receiver operating curve analysis, an optimal cut-off for high NLR (>3.5) in predicting overall survival (OS) was derived. Survival analysis was performed using the Kaplan-Meier method and multivariable Cox proportional regression. In patients with high NLR, estimated 5-year OS was 25% compared to 53% in those with low NLR. In multivariable analysis, high NLR, in addition to age ≥60 years, presence of B-symptoms and stage III/IV at diagnosis, was independently correlated with worse OS (HR 2.08; 95% CI 1.36 to 3.18; *p* = 0.0008) and progression-free survival (HR 1.66; 95% CI 1.11 to 2.46; *p* = 0.0128). A new prognostic index (NABS score) derived from these factors stratified patients into low (0), low-intermediate (1), high-intermediate (2) and high (3–4) risk subgroups, which were associated with 5-year OS of 76.5%, 55.7%, 29.2% and 0% respectively. In conclusion, high NLR is an independent prognostic marker and the NABS model can be used to risk-stratify NKTL patients.

## Introduction

Extranodal natural killer/T-cell lymphoma, nasal type (NKTL) is a rare but aggressive subtype of peripheral T-cell lymphoma (PTCL)^1^. The epidemiology of this entity has significant geographical variation, with a higher incidence in East Asian and Latin American populations but rare in North America and Europe^2^. Nonetheless, reports of NKTL in the West have been increasing steadily, possibly due to improved understanding and recognition of this disease entity^3^. While much remains unknown regarding the tumorigenesis of NKTL, recent studies have led to deeper insights of its clinical behavior and molecular pathobiology. NKTL classically manifests as a destructive lesion in the nasal cavity and upper aerodigestive tract, with the presence of extranasal disease characterized by more adverse clinical features and worse survival outcomes^4^. Most cases originate from NK cells while a minority are derived from T-cells^5^, and at the molecular level, we and others have shown that constitutive activation of the JAK/STAT signaling pathway plays an important role in promoting NKTL cell growth^6–8^.

Despite improvements in patient outcomes with the introduction of L-asparaginase-based chemotherapy regimens and modern radiotherapy in the management of NKTL, disease relapse rates remain substantial even for local disease and 5-year survival rates for advanced cases are a dismal 50%^9^. A recently described prognostic model (Prognostic Index of Natural Killer lymphoma, PINK) identified four risk factors—age greater than 60 years, stage III or IV disease, distant lymph node involvement and non-nasal type to be strongly associated with worse survival outcomes^10^. Notably, there is increasing evidence that peripheral blood indices of systemic inflammation may signify an aggressive phenotype in both solid and hematological malignancies^2,11,12^. Inflammation plays a pivotal role in the tumor microenvironment through the production and release of cytokines, which promotes tumorigenesis, angiogenesis, invasion and metastasis^13^. Mutagenic substances are also released by inflammatory cells, facilitating the development of further mutations that may provide cancer cells with a survival advantage^14^. In this respect, peripheral blood indices of systemic inflammation such as the neutrophil-lymphocyte ratio (NLR) has been consistently demonstrated to be prognostic in Hodgkin lymphomas^15^, B-cell lymphomas^16^, and subtypes of PTCL other than NKTL^12^. Therefore in this study, we aim to investigate the systemic inflammatory milieu in NKTL through the study of pre-treatment peripheral blood counts and explore its clinical prognostic relevance in these patients.

## Results

### Patient demographics

Table 1 shows the clinical characteristics of all 178 patients. The median age was 54.0 years (range 17.1 to 86.1 years) with 32 patients (18.0%) (over 60 years old. There was a male predominance (70.8%). One hundred and thirteen patients presented with early-stage disease (Ann-Arbor I or II), 155 had good performance status (ECOG score 0 or 1) and 74 had B symptoms at presentation. Table 2 shows the first line treatment modality patients received. One hundred and eight patients (60.7%) received chemotherapy, which included the following regimens: SMILE (n = 36), anthracycline-based (n = 20), gemcitabine-based (n = 12) and others (n = 40). Sixty patients (33.7%) received concurrent chemoradiation, 8 (4.5%) received radiation only while 2 (1.1%) received supportive care only.

**Table 1:** Clinicopathological features and NLR at diagnosis.

Characteristic (n)	Neutrophil-lymphocyte ratio at diagnosis (%)		*p*
	≤3.5	>3.5	
Total (178)	119 (66.9)	59 (33.1)	—
*Sex*			0.435
Male (126)	82 (65.1)	44 (34.9)	
Female (52)	37 (71.2)	15 (28.8)	
*Age at diagnosis (years)*			**0**.**0316**
≥60 (32)	18 (56.3)	14 (43.7)	
<60 (146)	101 (69.2)	45 (30.8)	
*Performance status (ECOG score)*			**0**.**0025**
0–1 (155)	110 (71.0)	45 (29.0)	
2–4 (23)	9 (39.1)	14 (60.9)	
*Ann Arbor stage*			0.255
I-II (113)	79 (69.9)	34 (30.1)	
III-IV (65)	40 (61.5)	25 (38.5)	
*Distal node involvement*			0.517
Yes (43)	27 (62.8)	16 (37.2)	
No (135)	92 (68.1)	43 (31.9)	
*B-symptoms*			0.263
Present (74)	46 (62.2)	28 (37.8)	
Absent (104)	73 (70.2)	31 (29.8)	
*LDH elevation*			**0**.**0197**
Yes (89)	52 (58.4)	37 (41.6)	
No (88)	66 (75.0)	22 (25.0)	
*EBV detection*			**0**.**0084**
Yes (65)	41 (63.1)	24 (36.9)	
No (71)	59 (83.1)	12 (16.9)	
*IPI risk group*			**0**.**0142**
1–2 (124)	90 (72.6)	34 (27.4)	
3–4 (54)	29 (53.7)	25 (46.3)	
*PINK score*			0.206
0–1 (117)	82 (70.1)	35 (29.9)	
2–4 (61)	37 (60.7)	24 (39.3)	
*Platelet-lymphocyte ratio*			<**0**.**0001**
>201 (66)	21 (31.8)	45 (68.2)	
≤201 (112)	98 (87.5)	14 (12.5)	
*Lymphocyte-monocyte ratio*			<**0**.**0001**
≤1.8 (46)	11 (23.9)	35 (76.1)	
>1.8 (132)	108 (81.8)	24 (18.2)	

Missing data: LDH (n = 1), EBV (n = 42).

**Table 2:** First line treatment modality received.

Treatment modality	n (%)		
	Overall (n = 178)	Singapore (n = 64)	Korea (n = 114)
Supportive care only	2 (1.1)	2 (3.1)	0 (0)
Radiation only	8 (4.5)	8 (12.5)	0 (0)
Concurrent chemoradiation	60 (33.7)	0 (0)	60 (52.6)
Chemotherapy	108 (60.7)	54 (84.4)	54 (47.4)
• Anthracycline-based	20 (11.2)	15 (23.4)	5 (4.4)
• Gemcitabine-based	12 (6.7)	12 (18.8)	0 (0)
• SMILE	36 (20.2)	4 (6.3)	32 (28.1)
• Others	40 (22.5)	23 (35.9)	17 (14.9)

Across the entire cohort, the values for NLR (median: 2.6, range: 0.7 to 24.5), platelet-lymphocyte ratio (PLR) (median: 158.4, range: 19 to 1708.9) and lymphocyte-monocyte ratio (LMR) (median: 2.8, range: 0.4 to 10.7) follow non-normal distributions (all *p* < 0.0001). Patients were dichotomized according to levels of NLR, PLR, LMR using optimized cut-offs to predict overall survival (OS) as derived from ROC curve analysis (>3.5, >201 and ≤1.8, respectively). The areas under the curve for NLR, PLR and LMR for OS were 0.622 (95% CI 0.546 to 0.693), 0.586 (95% CI 0.510 to 0.660), and 0.585 (95% CI 0.509 to 0.659), respectively.

### Clinicopathological correlates

**Figure 1:**
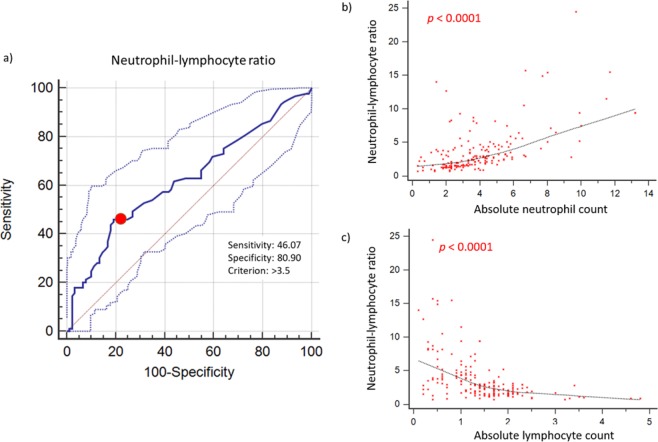
Derivation of NLR. (**a**) An optimal cut-off for high NLR (>3.5) in predicting overall survival and progression-free survival was determined using receiver operating curve analyses. (**b**) A positive correlation was demonstrated between NLR and absolute neutrophil counts (Spearman’s rho 0.571, 95% CI 0.463 to 0.662, p <0.0001), (**c**) while a converse correlation with absolute lymphocyte counts was found (Spearman’s rho −0.591, 95% CI −0.679 to −0.486, p <0.0001).

A subgroup of 59 patients (33.1%) had high NLR >3.5 at diagnosis. NLR >3.5 was significantly associated with age ≥ 60 years old (*p* = 0.0316), ECOG score ≥2 (*p* = 0.0025), elevated serum LDH (*p* = 0.0197), plasma Epstein-Barr virus (EBV) detection (*p* = 0.0084), IPI score ≥3 (*p* = 0.0142), PLR >201 (*p* < 0.0001) and LMR ≤1.8 (*p* < 0.0001) but not with sex, Ann-Arbor stage, distal node involvement, presence of B symptoms, and PINK score. A positive correlation was demonstrated between NLR and absolute neutrophil counts (Spearman’s rho 0.571, 95% CI 0.463 to 0.662, *p* < 0.0001), while a converse correlation with absolute lymphocyte counts was found (Spearman’s rho −0.591, 95% CI −0.679 to −0.486, *p* < 0.0001) (Fig. 1).

### Survival analyses

**Figure 2:**
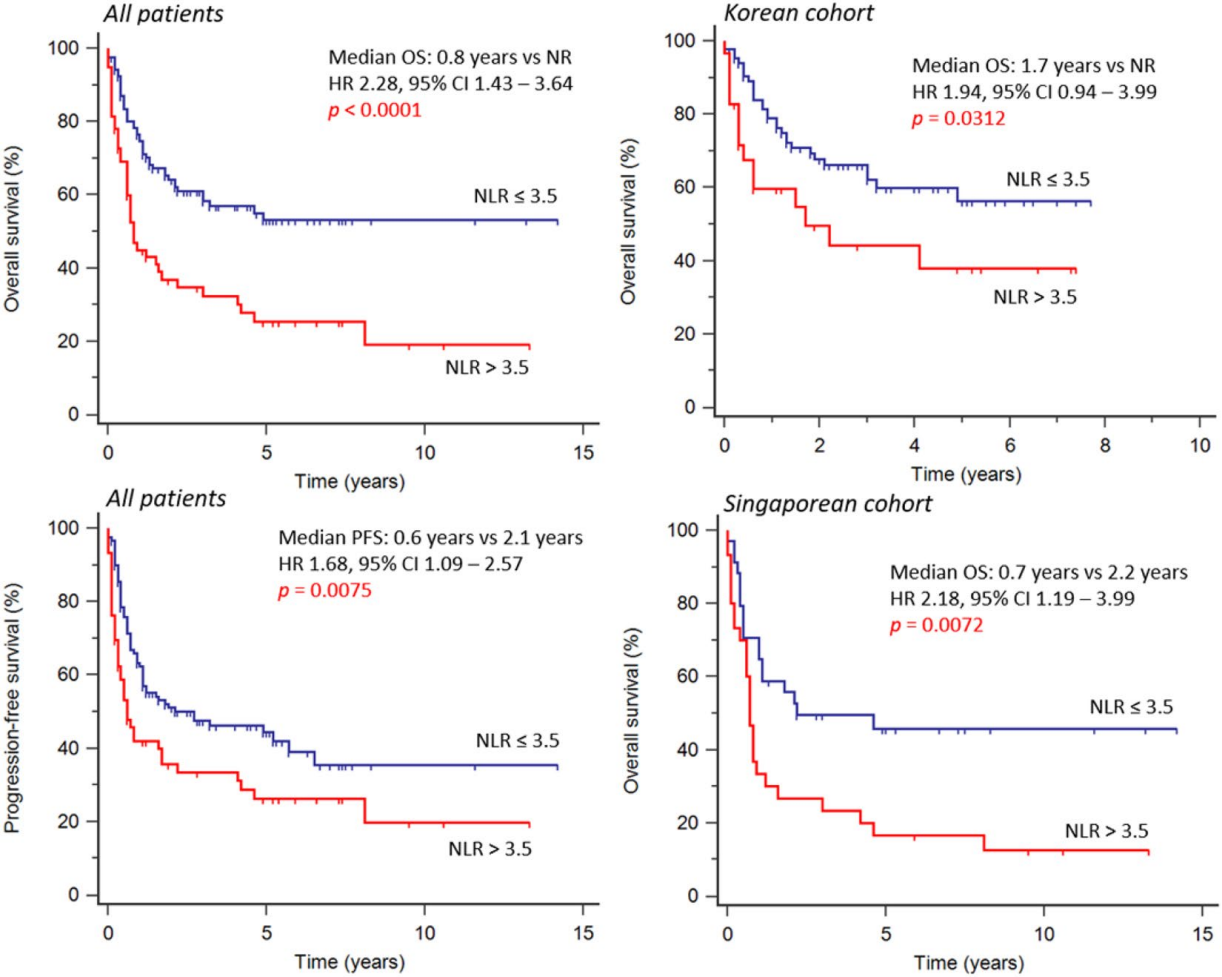
Survival outcomes stratified by NLR. High NLR (>3.5) was associated with worse overall survival and progression-free survival in analysis of the entire cohort. In subgroup analysis, high NLR was consistently associated with worse overall survival both in the Singaporean and Korean cohorts.

**Figure 3:**
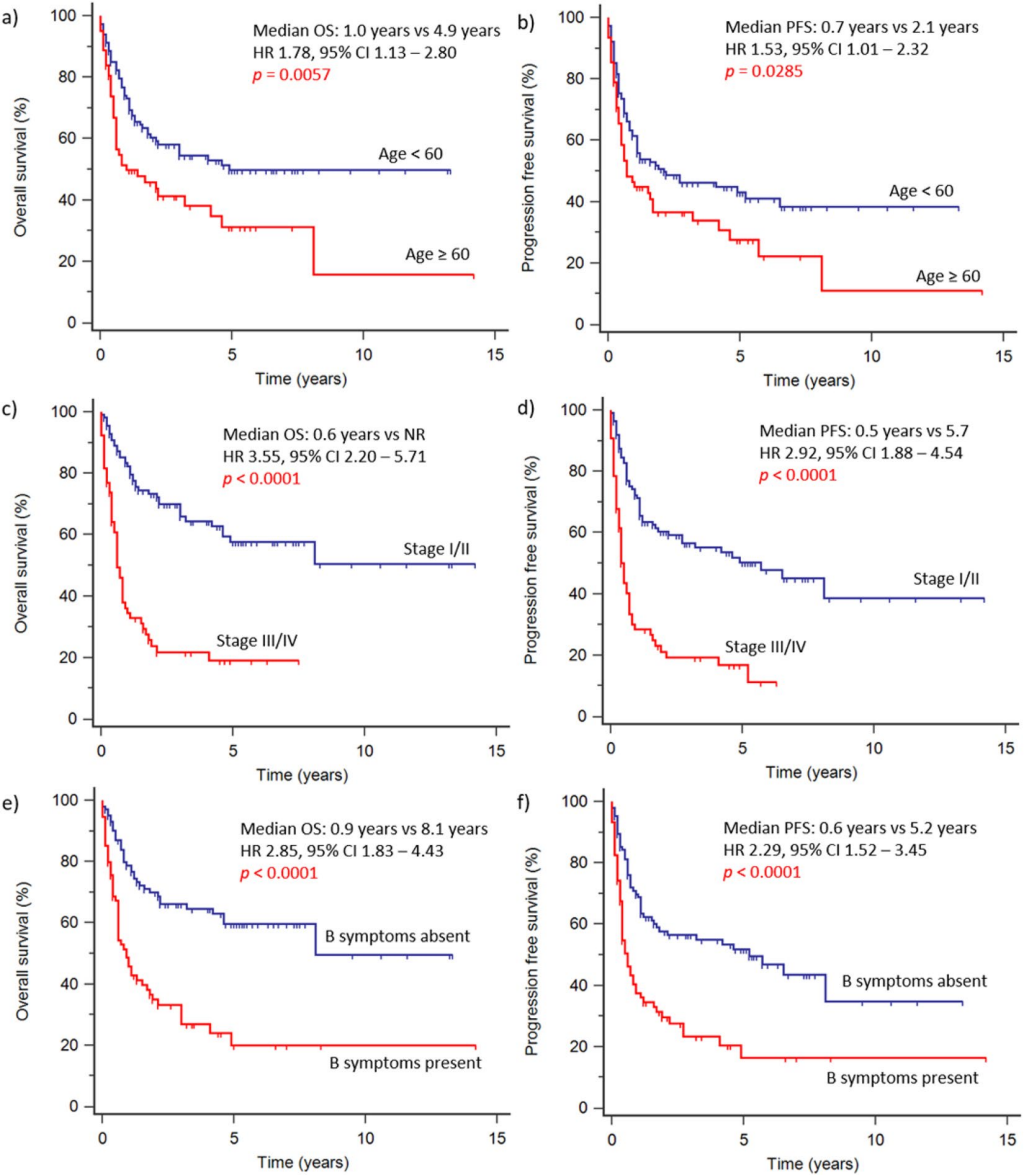
Survival outcomes stratified by age, Ann-Arbor staging and presence of B symptoms. Older age at diagnosis (≥60 years old), advanced Ann-Arbor staging (stage III/IV) and presence of B symptoms was associated with worse overall survival and progression-free survival.

At the time of analysis, 89 patients (50%) had died. The median follow-up time was 5.2 years. In the overall cohort, NLR >3.5 was associated with worse OS (HR 2.28, 95% CI 1.43 to 3.64, *p* < 0.0001) (Fig. 2). Median OS was 0.8 years in patients with NLR >3.5 and 2.1 years with NLR ≤3.5. Estimated 5-year OS was 53% in patients with low NLR and 25% in those with high NLR. Under univariable analysis (Table 3), the following factors at diagnosis were statistically significant predictors for OS: NLR >3.5, age ≥60 years old, ECOG score ≥2, advanced stage (III or IV), distal node involvement, presence of B symptoms, elevated serum LDH, PLR >201, LMR ≤ 1.8, PINK score ≥ 2 and IPI score ≥ 3. These factors were also statistically significant predictors for PFS, except for PLR. Multivariate analysis (Table 4) demonstrated that high NLR was a significant independent predictor for both OS (HR 2.08, 95% CI 1.36 to 3.18, *p* = 0.0008) and PFS (HR 1.66, 95% CI 1.11 to 2.46, *p* = 0.0128). In addition, age (HR 1.70, 95% CI 1.11 to 2.62, *p* = 0.0156), advanced stage (HR 3.06, 95% CI 1.94 to 4.82, *p* <0.0001) and presence of B symptoms (HR 2.35, 95% CI 1.49 to 3.71, *p* = 0.0002) were also significant independent predictors of OS (Fig. 3). For PFS, advanced stage (HR 2.63, 95% CI 1.74 to 3.98, *p* <0.0001) and presence of B symptoms (HR 1.89, 95% CI 1.25 to 2.85, *p* = 0.0024) were significant independent predictors. In subgroup analysis, NLR >3.5 was a significant predictor for both poor OS (HR 2.18, 95% CI 1.19 to 3.99, *p* = 0.0072) and PFS (HR 1.74, 95% CI 0.98 to 3.11, *p* = 0.0460) in the Singaporean cohort. For the Korean cohort, high NLR was associated with poor OS (HR 1.94, 95% CI 0.94 to 3.99, *p* = 0.0312), but not PFS (HR 1.30, 95% CI 0.69 to 2.45, *p* = 0.372).

**Table 3:** Univariable survival analysis for entire cohort.

Characteristic	Progression-free survival		Overall survival	
	HR (95% CI)	*p*	HR (95% CI)	*p*
Sex	0.97	0.8779	1.15	0.5579
(male *vs* female)	0.63 to 1.48		0.73 to 1.81	
Age at diagnosis	1.53	**0.0285**	1.78	**0.0057**
(≥60 *vs* <60 years)	1.00 to 2.32		1.13 to 2.80	
ECOG score	2.57	**0.0001**	3.44	**<0.0001**
(2–4 *vs* 0–1)	1.29 to 5.11		1.58 to 7.49	
Stage	2.92	**<0.0001**	3.55	**<0.0001**
(III-IV vs I-II)	1.88 to 4.54		2.20 to 5.71	
Distal node involved	2.21	**0.0001**	2.71	**<0.0001**
(Yes *vs* No)	1.34 to 3.64		1.58 to 4.66	
B-symptoms	2.29	**<0.0001**	2.85	**<0.0001**
(Present *vs* absent)	1.52 to 3.45		1.83 to 4.43	
LDH elevation	2.15	**0.0001**	2.51	**<0.0001**
(Yes *vs* No)	1.45 to 3.17		1.65 to 3.83	
NLR at diagnosis	1.68	**0.0075**	2.28	**<0.0001**
(>3.5 *vs* ≤3.5)	1.09 to 2.57		1.43 to 3.64	
PLR at diagnosis	1.37	0.1022	1.81	**0.0037**
(>201 *vs* ≤201)	0.91 to 2.05		1.17 to 2.81	
LMR at diagnosis	1.55	**0.0329**	1.84	**0.0047**
(≤1.8 *vs*>1.8)	0.98 to 2.46		1.12 to 3.02	
PINK score	2.95	**<0.0001**	3.80	**<0.0001**
(2–4 vs 0–1)	1.87 to 4.65		2.31 to 6.23	
IPI risk group	2.85	**<0.0001**	3.40	**<0.0001**
(3–4 *vs* 1–2)	1.78 to 4.57		2.04 to 5.67

**Table 4:** Multivariate survival analysis for entire cohort.

Characteristic	Progression-free survival		Overall survival	
	HR (95% CI)	*p*	HR (95% CI)	*p*
Age at diagnosis	—	—	1.70	**0.0156**
(≥60 *vs* <60 years)			1.11 to 2.62	
Stage	2.63	**<0.0001**	3.06	**<0.0001**
(III-IV vs I-II)	1.74 to 3.98		1.94 to 4.82	
B-symptoms	1.89	**0.0024**	2.35	**0.0002**
(Yes *vs* No)	1.25 to 2.85		1.49 to 3.71	
NLR at diagnosis	1.66	**0.0128**	2.08	**0.0008**
(>3.5 vs ≤3.5)	1.11 to 2.46		1.36 to 3.18	

Covariates included age at diagnosis, stage, B-symptoms, distal node involvement, ECOG performance status, sex, LDH elevation, NLR, PLR and LMR.

**Figure 4:**
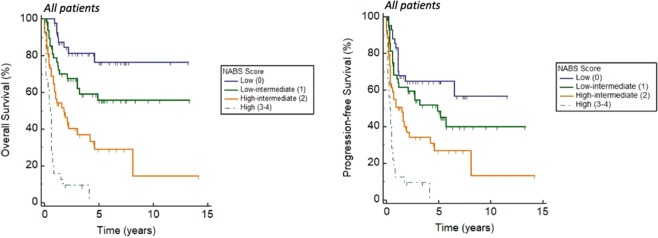
Overall and progression-free survival stratified by NABS score. Patients were risk-stratified based on their NABS score into low (0), low-intermediate (1), high-intermediate (2) and high (3–4) risk subgroups, which were associated with 5-year OS of 76.5%, 55.7%, 29.2% and 0%, and 2-year PFS of 64.8%, 59.5%, 37.1% and 9.4% respectively.

A prognostic index was then derived from the 4 independent prognostic factors for OS, namely NLR, age ≥60, presence of B symptoms and advanced stage (NABS score). Patients were then further risk-stratified based on their score into low (0), low-intermediate (1), high-intermediate (2) and high (3–4) risk subgroups, which were associated with 5-year OS of 76.5%, 55.7%, 29.2% and 0%, and 2-year PFS of 64.8%, 59.5%, 37.1% and 9.4% respectively for the entire cohort (*p* <0.0001) (Fig. 4). The stratification into subgroups was significantly associated with both OS and PFS in both the Korean and Singaporean cohorts (*p* <0.0001).

### Gene set enrichment analysis

**Figure 5:**
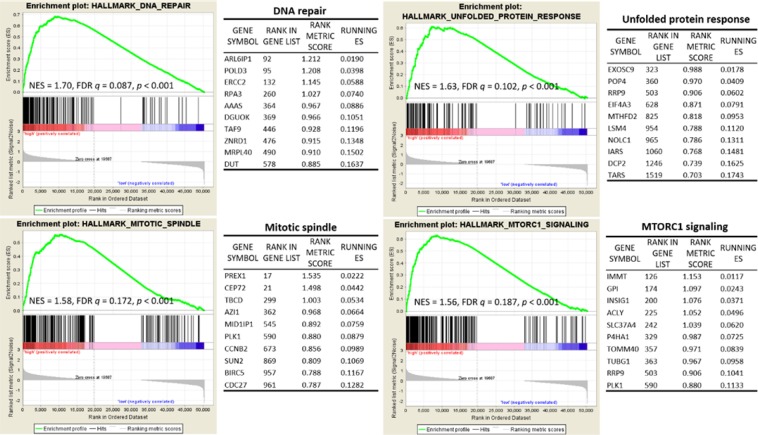
Gene set enrichment analysis (GSEA). Using the Hallmark gene set, upregulation of genes involved in DNA repair, unfolded protein response, mitotic spindle, and MTORC1 signaling were significantly enriched in cases with elevated NLR. The top 10 genes in each pathway are shown in the corresponding tables.

In an exploratory analysis, tumor gene expression profiles available from 8 patients were examined for differences between those with high NLR and those with low NLR (4 with NLR> 3.5 and 4 with NLR <3.5) by gene set enrichment analysis (GSEA). Using the Hallmark gene set, up-regulation of genes involved in DNA repair (NES = 1.70, FDR *q* = 0.087, *p* <0.001), genes associated with the unfolded protein response (NES = 1.63, FDR *q* = 0.102, *p* <0.001), genes important for mitotic spindle assembly (NES = 1.58, FDR *q* = 0.172, *p* <0.001) as well as those up-regulated through activation of the mTORC1 complex (NES = 1.56, FDR *q* = 0.187, *p* <0.001) were significantly enriched in cases with elevated NLR (Fig. 5).

## Discussion

Our current study demonstrates that a subset of NKTL patients possesses an elevated systemic inflammatory status as measured by peripheral blood indices. This inflammatory phenotype had adverse prognostic implications, as indicated by poor survival outcomes in these patients. Neutrophils, as part of the innate immune system, are a proxy of ongoing systemic inflammation. Increases in neutrophil counts have been shown to precede oncogenesis in some cases, while in others, malignant change stimulates the inflammatory process instead^17^. In other words, inflammation may facilitate survival of cancer cells, while cancer cells in turn release pro-inflammatory cytokines, augmenting each other in a perpetual spiral. The inflammatory tumor microenvironment then facilitates hallmark functions of cancer cells, such as proliferation, angiogenesis, invasion and metastasis^13^. Furthermore, neutrophils may contribute to functional suppression of lymphocytes^18,19^, facilitating tumor immune escape and promotion of metastasis^20^. Given these contrasting roles of neutrophils and lymphocytes in the immune regulation of cancer cells, an elevated NLR may set up an optimal immune microenvironment to promote tumor dissemination, thereby resulting in poor patient outcomes.

Previous studies in lymphomas have consistently shown that high NLR at diagnosis is a prognostic of poorer outcomes. In nodular sclerosis Hodgkin lymphoma, NLR> 6 was associated with worse OS (HR 1.54, 95% CI 1.03 to 2.29, *p* = 0.034) and PFS (HR 1.65, 95% CI 1.25 to 2.18, *p* = 0.001)^15^; in B-cell lymphomas, NLR ≥ 3.5 was associated with worse OS and PFS^16^; in subtypes of PTCL other than NKTL, NLR ≥ 4 was associated with worse OS and PFS as well^12^. Furthermore, lymphopenia has been associated with a worse outcome in NKTL^21,22^. In one study on early-stage NKTL, PLR > 185 was found to be associated with worse OS (HR 1.77, 95% CI 1.10 to 2.87, *p* = 0.02)^23^. A recent study by Zhou *et al*. showed that both lymphopenia and an elevated derived NLR were prognostic for worse OS and PFS^24^, supporting our findings.

Our own results showed that high NLR, but not PLR or LMR, was independently associated with worse PFS and OS. Taken together, it appears that the NLR may be a reliable prognostic biomarker in lymphoma. The NABS prognostic model represents a cost-effective means to easily classify patients into distinct subgroups with significantly different prognosis, which can be used to develop risk-adapted treatment approaches for patients with NKTL.

Interestingly, our results in a small subset of patients showed that NKTL associated with an elevated peripheral blood NLR and poor patient outcomes demonstrated higher expression levels of DNA repair genes. Previously, Bald *et al*. showed that ultraviolet radiation-induced DNA damage leads to pro-metastatic neutrophilic inflammation, stimulating perivascular invasion and metastasis in melanomas^25^. Though preliminary, our findings of upregulated pro-proliferative pathways including the mitotic spindle assembly as well as mTORC1^26^ further support a more aggressive phenotype in this group of NKTL.

Our current study does have certain limitations. Firstly, as a retrospective study, information on EBV status and beta 2-microglobulin levels were not collected for all patients, limiting the ability to take into these potential confounders, as they have been found to be independent predictors of poorer outcome in patients with NKTL^10,27,28^. Secondly, both patient cohorts in our study received heterogenous treatments (Table 2), which may have affected their prognosis. However, the observation that NLR and NABS score was consistently prognostic in both the Singaporean and Korean patients suggests that its prognostic impact is probably independent of treatment regimen. Nonetheless, future prospective studies would be necessary to confirm our findings, especially in a larger cohort treated with non-anthracycline and L-asparaginase containing regimens, the mainstay of current treatment.

## Conclusions

In patients with NKTL, peripheral blood NLR at time of diagnosis is an inexpensive clinical prognostic marker that can be added to current prognostic models to refine risk stratification. The NABS score represents a new prognostic model for risk-stratification, although further validation in a prospective study of an independent cohort is required.

### Ethics approval and consent to participate

Written consent for use of biospecimens and clinical data were obtained in accordance with the Declaration of Helsinki. Tissue collection and consent protocols were performed as part of the Singapore Lymphoma Study and were under approval from the SingHealth Centralized Institution Review Board as well as the Institutional Review Board of Samsung Medical Center.

## Patients and Methods

### Study cohort

Retrospective review of clinical data of patients with histologically-proven NKTL seen at the National Cancer Centre Singapore (NCCS), Singapore General Hospital (SGH) and Samsung Medical Center (SMC) from 1993 to August 2016 was performed. A total of 178 patients who had available pre-treatment peripheral blood neutrophil and lymphocyte counts at the time of diagnosis were included in the final analysis^29^. Those with ongoing active infections, concurrent hematological disorders or receipt of corticosteroids at the time of blood draw were excluded. Clinicopathological information available included age, sex, ethnicity, presence of B symptoms at diagnosis, ECOG performance scores, LDH levels and Ann Arbor stage. National Registration Identification Cards were used to verify patient demographics. All histological parameters were reviewed by expert haematolymphoid pathologists. Patient characteristics are summarized and stratified according to NLR in Table 1.

### cDNA synthesis, gene expression profiling and gene set enrichment analysis

Informed consent for use of biospecimens were obtained in accordance with the Declaration of Helsinki. Total RNA was extracted using TRIzol (Invitrogen, Carlsbad, CA, USA) and purified with RNeasy Mini Kit (Qiagen, Hilden, Germany) according to manufacturer’s instructions. The integrity of RNA was determined by electrophoresis using the 2100 Bioanalyzer (Agilent Technologies, Palo Alto, CA, USA). Total RNA (500 ng) was reverse transcribed with iScript cDNA Synthesis Kit (Bio-Rad, Hercules, CA, USA). Eight available NKTL samples were analysed by RNA-seq to estimate the transcript abundance of each protein-coding gene. mRNA sequencing was performed on Illumina HiSeq2000 (Illumina, San Diego, CA, USA) using the standard Illumina RNA-seq protocol. The reads were aligned to the human genome hg19 assembly using STAR^30^. Transcript abundance estimation was performed using RSEM^31^. For each gene, read counts are represented as “transcripts per million” (TPM) and are normalized for both sequencing depth and gene length. Gene set enrichment analysis (GSEA) was performed using the Molecular Signatures Database (MSigDB) Hallmark gene set^32^. The permutation type was based on phenotype, using a total of 1000 iterations. A gene set is considered to be significantly enriched if its Normalized Enrichment Score (NES) has a False Discovery Rate (FDR) q-value below 0.25 and nominal p-value <0.001.

### Statistical analysis

NLR and PLR were derived by dividing the absolute neutrophil counts and absolute platelet counts by the absolute lymphocyte counts, respectively, while the LMR was derived by dividing the absolute lymphocyte counts by the absolute monocyte counts. Normality of distribution of NLR, PLR and LMR were determined using the Kolmogorov-Smirnov test.

In previous studies, empirical cutoffs for NLR had been derived using heterogeneous methods^33^. These studies had not demonstrated any consistent or validated cut-off values, and were highly variable (ranging from 2.1 to 5.5). Given the lack of a discriminatory method to dichotomize NLR based on biological criteria^34,35^, we thus selected the receiver operative characteristic (ROC) curve analysis as the most objective statistical method for our study. ROC curve analysis via the method of DeLong *et al*.^36^ was then used to derive the optimal cut-off values for each parameter as a univariable predictor of overall survival (OS). The area under the curve (AUC) was 0.622 (95% CI 0.546 to 0.693). The sensitivity and specificity were 46.1% (95% CI 35.4% –57.0%) and 80.9% (95% CI 71.2% –88.5%) using a cut-off value of 3.5. The Hosmer-Lemeshow goodness-of-fit test indicated that the model was well-calibrated (chi-square 6.0144, *p* = 0.6456). To address the possibility of overfitting causing optimism regarding the model’s performance, we performed internal validation using a bootstrap validation algorithm with 1000 repetitions. The bootstrap validation obtained an AUC estimate of 0.620, indicating negligible overfitting of the model.

OS, the primary survival endpoint, was determined by the interval between date of diagnosis to the date of death from any cause; while progression-free survival (PFS), the secondary survival endpoint, was determined by the interval between date of diagnosis to date of relapse or date of death from any cause, whichever comes earliest. Survival was censored at the date of the last follow-up for survivors. Comparisons of the frequencies of categorical variables were performed using Pearson’s Chi-squared tests. Correlation analysis between continuous variables were evaluated by Spearman’s rho. Kaplan-Meier analyses were conducted to identify statistically significant univariable predictors of OS, and represented by hazard ratios (HR) and 95% confidence intervals (95% CI). Multivariate Cox regression model via a stepwise procedure was employed to determine independence of significant factors identified on univariable analysis. All statistical analyses were conducted assuming a two-sided test with significance level of 0.05 unless otherwise stated, and performed using MedCalc for Windows, version 18.2.1 (MedCalc Software, Ostend, Belgium).

## Data Availability

All data generated or analysed during this study are included in this published article. The dataset analyzed is available from the corresponding author on appropriate request.
